# Zika Virus Requires the Expression of Claudin-7 for Optimal Replication in Human Endothelial Cells

**DOI:** 10.3389/fmicb.2021.746589

**Published:** 2021-09-20

**Authors:** Jim Zoladek, Vincent Legros, Patricia Jeannin, Maxime Chazal, Nathalie Pardigon, Pierre-Emmanuel Ceccaldi, Antoine Gessain, Nolwenn Jouvenet, Philippe V. Afonso

**Affiliations:** ^1^Unité Épidémiologie et Physiopathologie des Virus Oncogènes, Institut Pasteur, Centre National de la Recherche Scientifique UMR 3569, Université de Paris, Paris, France; ^2^VetAgro Sup, Centre International de Recherche en Infectiologie (CIRI), Lyon, France; ^3^Unité Signalisation Antivirale, Institut Pasteur, Centre National de la Recherche Scientifique UMR 3569, Paris, France; ^4^Groupe Arbovirus, Unité Environnement et Risques Infectieux, Institut Pasteur, Paris, France

**Keywords:** flavivirus, Zika virus, claudin, endothelial cells, infection, blood-brain barrier

## Abstract

Zika virus (ZIKV) infection has been associated with a series of neurological pathologies. In patients with ZIKV-induced neurological disorders, the virus is detectable in the central nervous system. Thus, ZIKV is capable of neuroinvasion, presumably through infection of the endothelial cells that constitute the blood-brain barrier (BBB). We demonstrate that susceptibility of BBB endothelial cells to ZIKV infection is modulated by the expression of tight-junction protein claudin-7 (CLDN7). Downregulation of CLDN7 reduced viral RNA yield, viral protein production, and release of infectious viral particles in several endothelial cell types, but not in epithelial cells, indicating that CLDN7 implication in viral infection is cell-type specific. The proviral activity of CLDN7 in endothelial cells is ZIKV-specific since related flaviviruses were not affected by CLDN7 downregulation. Together, our data suggest that CLDN7 facilitates ZIKV infection in endothelial cells at a post-internalization stage and prior to RNA production. Our work contributes to a better understanding of the mechanisms exploited by ZIKV to efficiently infect and replicate in endothelial cells and thus of its ability to cross the BBB.

## Introduction

Zika virus (ZIKV) is an arbovirus from the *Flaviviridae* family, which was first isolated in Uganda in 1947 (Dick et al., 1952). It was initially disregarded as only sporadic and mild cases were reported. With the virus outbreaks in the Pacific Islands and the Americas in 2015, ZIKV received widespread attention. These outbreaks were associated with neurological pathologies, such as Guillain-Barré syndrome (Cao-Lormeau et al., 2016), congenital microcephaly (Cauchemez et al., 2016), meningoencephalitis (Carteaux et al., 2016), and myelitis (Mecharles et al., 2016). During ZIKV-induced neurological disorders, the virus is detected in the central nervous system (CNS), and viral RNA can be amplified from the cerebrospinal fluid of infected patients (Roze et al., 2016).

In normal condition, the CNS is protected from blood-borne pathogens by the blood-brain barrier (BBB), a structure composed of astrocytes, pericytes, microglia cells, a basal membrane, and a continuous endothelium with tight junctions (Ballabh et al., 2004; Obermeier et al., 2016). In order to penetrate the CNS, ZIKV must cross the BBB. ZIKV infection induces BBB disruption in the later stages of the disease (Leda et al., 2019), as evidenced by fibrinogen diffusion into the CNS parenchyma (Panganiban et al., 2020) and neuroinvasion of infected monocytes (Ayala-Nunez et al., 2019; de Carvalho et al., 2019). However, during early ZIKV infection stages, the BBB remains intact. This is also the case during infection with other flaviviruses (Mustafa et al., 2019), where neuroinvasion occurs by transcytosis or productive infection of endothelial cells (Bramley et al., 2017; Mladinich et al., 2017; Papa et al., 2017; Cle et al., 2020).

Zika virus has a broad tropism for human cell types. ZIKV enters cells by endocytosis in a pH-dependent manner. The factors favoring ZIKV entry is not well defined. Adsorption of the virus at the cell surface is mediated through heparan sulfate proteoglycans (HSPG) and C-type lectin receptors interaction (Hamel et al., 2015; Kim et al., 2017). Endocytosis might involve apoptotic mimicry, as previously suggested for the related dengue virus (DENV; Amara and Mercer, 2015). *In vitro*, ZIKV endocytosis is mediated through recognition of phosphatidylserines present on the viral envelope by members of the TAM family (i.e., TYRO3, AXL, and MER) or TIM-1 receptor (Hamel et al., 2015; Meertens et al., 2017; Dejarnac et al., 2018). Although *in vitro AXL* depletion or the use of anti-AXL antibodies rendered cells resistant to infection (Liu et al., 2016; Meertens et al., 2017), *in vivo AXL* (or members of the TAM family) knock-out in IFNAR^−/−^ mice had no significant effect on these mice susceptibility to ZIKV infection (Hastings et al., 2017; Wang et al., 2017). The current model suggests that the AXL-dependent signaling inhibits the antiviral interferon (IFN) response, thereby promoting viral replication (Chen et al., 2018). Other factors have been proposed to act as cell-type specific receptors for ZIKV, such as, for example, integrin αVβ5 and NCAM1 in neural stem cells (Srivastava et al., 2020; Wang et al., 2020).

Claudins (CLDNs) are a large family of transmembrane proteins involved in cell-to-cell or cell-to-substrate interactions (Krause et al., 2008). They participate in the formation of tight junctions and are therefore key components of physiological barriers. Upon flavividae infection, BBB and TJ are often disrupted: infection with West Nile virus and classic swine fever virus both lead to claudin-1 degradation (Medigeshi et al., 2009; Roe et al., 2012; Wang et al., 2021) and Japanese encephalitis virus infection induces the degradation of claudin-5 (Chang et al., 2015). Claudins can also directly participate in viral infection (Colpitts and Baumert, 2017). Within the *Flaviviridae* family, dengue viruses use CLDN1 as a receptor in hepatocytes (Coller et al., 2009; Gao et al., 2010) and hepatitis C virus (HCV) exploits CLDN1, 6, and 9, as receptors in hepatocytes (Evans et al., 2007; Meertens et al., 2008).

Herein, we studied the susceptibility of BBB endothelial cells to ZIKV infection and identified CLDN7 as a key proviral factor in endothelial cells.

## Materials and Methods

### Cell Lines

Human kidney epithelial HEK 293T (ATCC, CRL-11268), human lung epithelial A549 (ATCC, CCL-185), and African green monkey kidney epithelial Vero E6 (ATCC, CRL-1586) cells were maintained in high-glutamine and high-glucose DMEM (Thermo fisher) supplemented with 10% fetal bovine serum (FBS; Thermo fisher), 100U/ml penicillin and 100μg/ml streptomycin (Thermo fisher). *Aedes albopictus* C6/36 (ATCC, CRL-1660) cells were maintained in Leibovitz’s L-15 medium (Thermo fisher) supplemented with 10% FBS. Transformed human bone marrow endothelial cells TrHBMEC (Schweitzer et al., 1997), human cerebral microvascular endothelial cells (hCMEC/D3; Merck, SCC066), and primary human umbilical vein endothelial cells HUVEC (ATCC, CRL-1730) cells were maintained in complete EndoGRO Basal Medium (Merck) supplemented with 5% FBS, 0.2% EndoGRO-LS supplement, 5ng/ml rhEGF, 10mM l-glutamine, 1μg/ml hydrocortisone hemisuccinate, 0.75U/ml heparan sulphate, and 50μg/ml ascorbic acid (Merck). Alternatively, hCMEC/D3 cells have been maintained in EBM-2 basal medium (Lonza) supplemented with 5% FBS, 1.5μM hydrocortisone (Merck), 5μg/ml ascorbic acid (Merck), 1% chemically defined lipid concentrate (Thermo fisher), 1ng/ml human bFGF (Merck), 10μM HEPES (Thermo fisher), and antibiotics. This culture condition was used when compared to EndoGRO media. All cells were kept at 37°C with 5% CO_2_ except for C6/36 which were kept at 28°C with no CO_2_.

### Viruses

Two strains of ZIKV were used in this study: the AF/1991/HD78788 strain (GenBank: KF383039) obtained in Senegal in 1991, which belongs to the African lineage, and the Brazil/2016/INMI1 strain (GenBank: KU991811) isolated during the 2016 outbreak in Brazil, which belongs to the Asian lineage. Japanese encephalitis virus JEV/G3/RP-9 (Liang et al., 2009) and yellow-fever vaccine strain YFV/17D (GenBank: MG051217) were used as controls, as they are closely related to ZIKV. Virus stocks were produced in C6/36 cells. All infections were performed in 2% FBS media in BSL-3 facilities.

### Gene Knockdown

Gene knockdown was performed using lentiviral vectors carrying shRNA. To produce these lentiviral vectors, HEK 293T cells were co-transfected with psPAX2 (encoding HIV Gag/Pol, 4.68μg; Addgene: 12260), pMD2.G [vesicular stomatitis virus G protein (VSV-G), 2.52μg; Addgene: 12259], and pLKO.1 encoding the shRNA (TRC-Hs1.0 library, 9μg, GE Dharmacon). Transfection was performed using LipoD-293 transfection reagent (Sinagen) according to the manufacturer’s recommendations. After 72h, supernatants were collected, centrifuged, and stored at −80°C.

Cells were exposed for 2h to the lentivirus-containing medium, and gene expression was allowed for 48h then transduced cells were selected with 1μg/ml Puromycin (Merck) for 2weeks. The obtained culture is thus a mix population of transduced cells.

### Microarray Analysis

Biotinylated single strand cDNA targets were prepared, starting from 250ng of total RNA, using the Ambion WT Expression Kit (Cat # 4411974) and the Affymetrix GeneChip® WT Terminal Labeling Kit (Cat # 900671) according to Affymetrix recommendations. Following fragmentation and end-labeling, 3μg of cDNAs were hybridized for 16h at 45°C on *GeneChip*® *Human Gene 2.0 ST arrays* (Affymetrix) interrogating over 400,000 RefSeq transcripts and ~11,000 LncRNAs. The chips were washed and stained in the GeneChip® Fluidics Station 450 (Affymetrix) and scanned with the GeneChip® Scanner 3000 7G (Affymetrix) at a resolution of 0.7μm. Raw data (CEL Intensity files) were extracted from the scanned images using the Affymetrix GeneChip® Command Console (AGCC) version 4.1.2. CEL files were further processed with Affymetrix Expression Console software version 1.4.1 to calculate probe set signal intensities using Robust Multi-array Average (RMA) algorithms with default settings. Data have been loaded into Array Express, accession number E-MTAB-10813.

### Gene Expression Analysis

Cellular RNA was extracted using the RNeasy Plus Mini Kit (Qiagen), quantified (Nanodrop ND-1000) and 500ng was reverse-transcribed using the SuperScript II Reverse Transcriptase (Thermo fisher) and random hexamer primers (Eurobio) with the following cycle: 2min at 95°C; 90min at 42°C; and 15min at 70°C (GeneAmp PCR System 9700, Applied Biosystems). SYBR Green RT-qPCR was performed on 25ng of complementary DNA with iTaq Universal SYBR Green Supermix (Biorad) using primers listed in Supplementary Table 1 with the following protocol: 15min at 95°C followed by 40cycles consisting of 15s at 95°C, 20s at 60°C, and 30s at 72°C (Mastercycler ep Realplex Thermal Cycler, Eppendorf). The melting curve was also obtained to control the amplified product. Relative expression was analyzed using the −∆∆*C*_T_ method.

Functional enrichment analysis of the differentially expressed genes was performed using FunRich 3.1.3 (Pathan et al., 2015).

### Western Blot Analysis

Cells were lysed using RIPA buffer (Merck) containing protease inhibitor cocktail (Merck) for 15min on ice. Cellular debris were not pelleted since claudins often accumulate in the insoluble fraction. Whole cell lysates were heat-denatured 70°C for 10min in LDS Sample Buffer and 500mM DTT (Novex). Proteins were resolved on NuPAGE 4–12% Bis Tris gel (Novex) in MOPS buffer and were transferred to nitrocellulose membranes (Trans Blot Turbo, Biorad). Blocking followed by immunoblotting was performed on an iBind Flex Western Device (Thermo fisher) using antibodies listed in Supplementary Table 2 according to the manufacturer’s recommendations.

### Viral Titration Assay

Supernatants were sequentially diluted 10-fold and inoculated onto Vero E6 monolayers in 24-well plates. After 1h incubation, 2% carboxymethyl cellulose (CMC; Merck) overlay containing 2% FBS culture medium was added to each well. For foci-formation assays, plates were then incubated at 37°C for 72h. Following the incubation, CMC overlay was removed and a PBS – paraformaldehyde 4% (Electron Microscopy Sciences) solution was added into each well and incubated at room temperature for 15min. Fixation solution was removed and plates were washed three times with PBS. Cells were then permeabilized with PBS – Triton 0.2% for 3min, washed three times, and incubated in blocking buffer (PBS – Tween 20 0.1% – FBS 1%) for 30min followed by 2h incubation with pan-*Flavivirus* antibody 4G2 purified from the ATCC hybridoma. Plates were washed three times with PBS followed by an hour-long incubation with a secondary antibody conjugated to horseradish peroxidase (Biorad). Detection was achieved using the VIP Peroxidase Assay (Vector) according to the manufacturer’s recommendations. Titer is expressed as foci-forming units (FFU).

Alternatively, for plaque-formation assays, plates were incubated at 37°C for 5days after adding the CMC overlay. They were then washed and fixed as described earlier. Plates were stained with a 1% crystal violet solution (Merck). Titer is expressed as plaque-forming units (PFU).

### Replication Assay

Infected cells were washed with PBS, and cellular RNA was extracted using the Nucleospin 96 RNA kit (Macherey Nagel). One-step RT-qPCR was performed using the Power SYBR Green RNA-to-*C*_T_ kit (Thermo fisher) with the following protocol: 30min at 48°C, 10min at 95°C followed by 40 cycles consisting of 15s at 95°C, and 1min at 60°C (QuantStudio 6 Flex Real-Time PCR System, Applied Biosystems). The melting curve was also obtained. Genome equivalent (GE) concentration was assessed with the standard curve method using known concentrations of synthetic RNA fragments corresponding to the ZIKV *NS5* coding region and was normalized to GAPDH level.

### Binding and Internalization Assay

Cells were seeded in 2% FBS medium 24h prior to infection. Pre-chilled cells were incubated with ZIKV (multiplicity of infection – MOI 1 and 10) for 1h at 4°C to block the internalization of the viruses. The cells were then washed three times with cold PBS to remove unbound particles. Cell surface adsorbed ZIKV particles was analyzed by harvesting RNA at this step. ZIKV internalization was induced by adding warm medium and keeping the cells at 37°C for 2h. The cells were then treated with trypsin (Thermo fisher) to remove un-internalized particles, cells were pelleted, and RNA was harvested.

### Immunofluorescence

Cells were fixed with 4% paraformaldehyde (PFA; Sigma-Aldrich) for 30min at room temperature, permeabilized with 0.5% Triton X-100 in PBS, and then blocked with PBS containing 0.05% Tween and 5% BSA before incubation with the anti-NS1 17A12 antibody (Schul et al., 2007). Antibody labeling was revealed with Alexa Fluor 488-conjugated antibody. Coverslips were mounted on slides using ProLong Gold Antifade reagent with NucBlue solution (Invitrogen). Images were acquired with a Zeiss LSM 700 inverted confocal microscope.

### Quantification and Statistical Analysis

**Figure 1:**
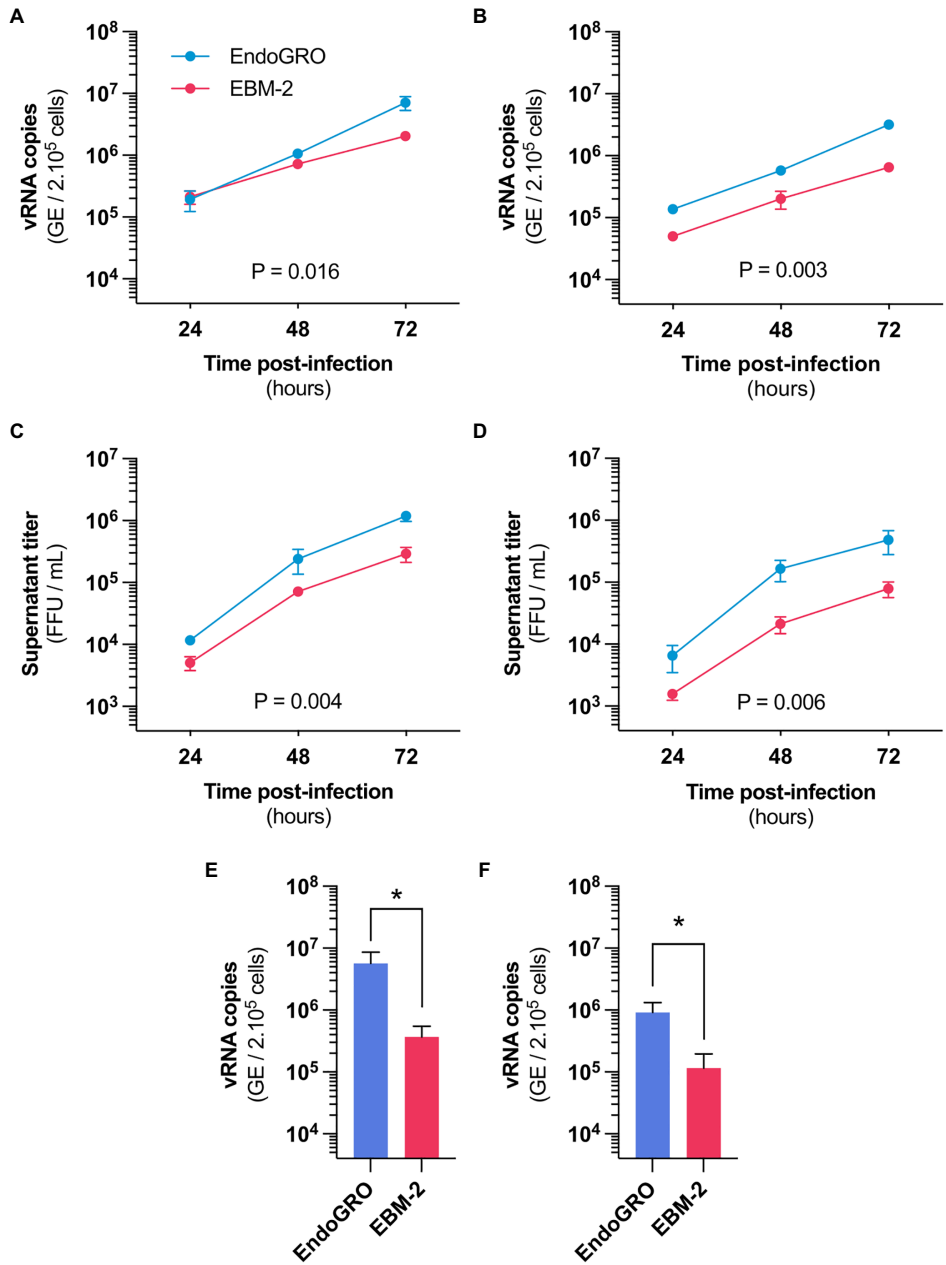
Culture medium alters hCMEC/D3 susceptibility to Zika virus (ZIKV) infection. **(A,B)** Time-course quantification of viral RNA through RT-qPCR in hCMEC/D3 cells cultured in EBM-2 (red) or EndoGRO (blue) and infected with the African HD78788 ZIKV strain. **(A)** Cells were infected at a MOI of 1 (*n*=4 independent experiments). **(B)** Cells were infected at a MOI of 0.1 (*n*=3 independent experiments). **(C,D)** Time-course quantification of viral production in the supernatant of hCMEC/D3 cells cultured in EBM-2 (red) or EndoGRO (blue) and infected with the African HD78788 ZIKV strain. **(C)** Cells were infected at a MOI of 1 (*n*=4 independent experiments). **(D)** Cells were infected at a MOI of 0.1 (*n*=3 independent experiments). **(E,F)** Quantification at 72h post-infection of viral RNA through RT-qPCR in hCMEC/D3 cells cultured in EndoGRO or EBM-2 and infected with the Brazilian INMI1 ZIKV strain. **(E)** Cells were infected at a MOI of 1 (*n*=3 independent experiments). **(F)** Cells were infected at a MOI of 0.1 (*n*=3 independent experiments). For each sample point, the experiment was performed in triplicate (each data point is the mean of three wells). Experiments were performed three or four times, as indicated. Data are presented as mean±SEM. Statistical significance was obtained using 2-Way ANOVA tests with Sidak’s *post-hoc* for **(A–D)** and Mann-Whitney test for **(E,F)**. Indicated value of *p* represents the difference for the “culture medium” factor. ^*^p≤0.05 (*post-hoc* corrected value of *p*). See also Supplementary Figure 1.

Specific statistical tests used to analyze experimental datasets are described in the respective figure legends. Graphical representations and statistical analyses were performed using GraphPad Prism version 8.2 for MacOS (GraphPad Software). Nonparametric tests were used, as we could not ascertain that the hypothesis for parametric tests were fulfilled. When comparing cells in the different media (Figure 1 and Supplemental Figures 1, 2), two-way ANOVA (followed by Sidak’s *post-hoc* tests) or the Mann-Whitney and Kruskal-Wallis tests were used (data are unpaired). When comparing the knockdown, we considered paired tests as a single batch of cells was transduced with the different shRNAs, and a new batch was used for each replicate. Therefore, two-way ANOVA (followed by Sidak’s *post-hoc* tests) or Friedman tests (followed by Dunn’s *post-hoc* tests) were used. Values of *p* indicated in figure legends corresponds to ANOVA result.

## Results

### The Susceptibility of hCMEC/D3 Cells to ZIKV Infection Depends on the Culture Medium

The human cerebral microvascular endothelial cell line hCMEC/D3 is a widely used model for *in vitro* human BBB studies (Weksler et al., 2005). Since ZIKV neuroinvasion relies on a productive infection of BBB endothelial cells (Liu et al., 2016; Mladinich et al., 2017; Papa et al., 2017; Richard et al., 2017; Cle et al., 2020), we examined the susceptibility to ZIKV infection of hCMEC/D3 cells.

We confirmed using RT-qPCR analysis that hCMEC/D3 cells can be productively infected with an African ZIKV strain (Figures 1A,B). The cells released infectious particles (Figures 1C,D), indicating that a complete viral replication cycle. Intriguingly, we found a difference in infection efficiency that was dependent on the cell culture medium, both in terms of viral RNA yield and production of infectious particles (Figure 1, EndoGRO vs. EBM-2). Differences were more marked at a lower MOI (Figures 1A,C vs. Figures 1B,D). Similar differences in viral susceptibility were observed with a Brazilian ZIKV strain (belonging to the Asian lineage), at 72h post-infection (Figures 1E,F).

Our first hypothesis was that factors present in the medium could interfere with viral infectivity. To test this, we suspended the viral stock in one or the other medium (or in DMEM as a control) and the inoculum was let to adsorb for 2h on hCMEC/D3 (previously cultured in EndoGRO). Cells were then washed and cultured in EndoGRO for 48h. RT-qPCR analysis performed at 48h post-infection revealed that the levels of intracellular viral RNA were similar in the three conditions (Supplementary Figure 1). This suggests that the culture media does not alter the cell-virus interaction *per se*. The differential susceptibility observed in the two culture conditions likely depend on differential gene expression.

We cultured hCMEC/D3 cells in EndoGRO or in EBM-2 prior to infection. Infection and subsequent culture were then performed in DMEM. We found that viral replication efficacy was dependent on the medium in which the cells were initially cultured (Supplementary Figure 2). This suggests that the cells, while being cultured in the different media, have acquired intrinsic differences to ZIKV infection susceptibility.

### Cells Cultured in EndoGRO Overexpress *Claudin*-7 When Compared to Cells Cultured in EBM-2

**Figure 2:**
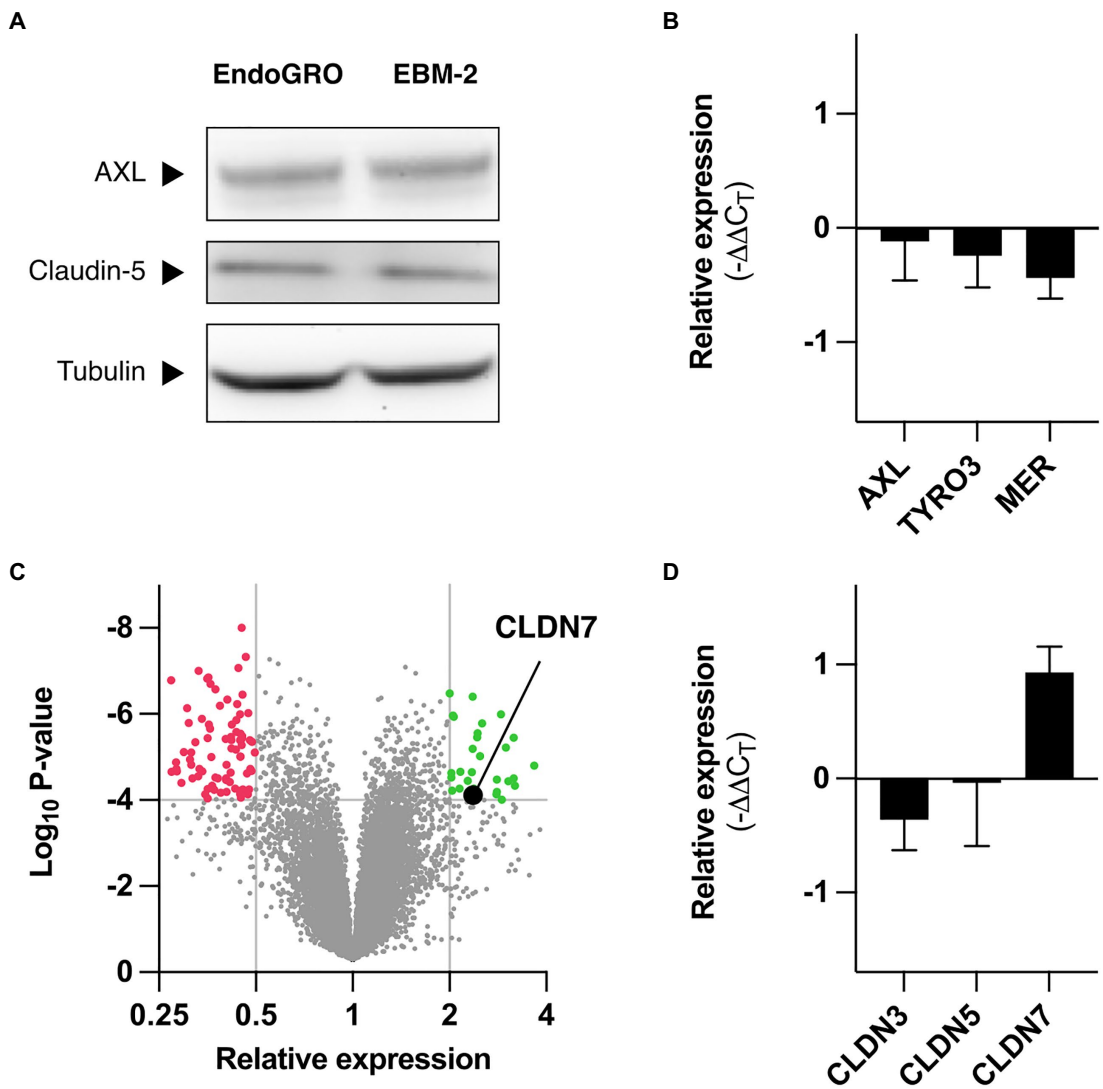
Differential gene expression in hCMEC/D3 depending on culture medium. **(A)** hCMEC/D3 cells were cultured in EndoGRO or EBM-2, and cell lysates were processed by western blot for studying expression levels of AXL and claudin-5. α-Tubulin serves as loading control. **(B)** hCMEC/D3 cells were cultured in EndoGRO or EBM-2, and mRNA levels for *AXL*, *TYRO3*, and *MERTK* in EndoGRO relative to EBM-2 were quantified (*n*=4 independent experiments). **(C)** hCMEC/D3 cells were cultured in EndoGRO or EBM-2, and mRNA was processed on an Affymetrix GeneChip Human Gene 2.0ST (*n*=3 independent experiments). The volcano plot presents the relative expression change of human genes in EndoGRO relative to EBM-2 and the statistical significance of the change. Thresholds (displayed as grey lines): *p*<10^–4^, relative expression <0.5 and relative expression >2. **(D)** hCMEC/D3 cells were cultured in EndoGRO or EBM-2, and mRNA levels for *CLDN3*, *CLDN5*, and *CLDN7* in EndoGRO relative to EBM-2 were quantified (*n*=4 independent experiments). Data are presented as mean±SEM.

First, we examined whether hCMEC/D3 cells cultured in the two media differentially expressed AXL and other members of the TAM family (i.e., TYRO3 and MER), which have been found to regulate endothelial susceptibility to ZIKV (Liu et al., 2016; Richard et al., 2017). Western blot analysis revealed that AXL expression remained unchanged in either medium (Figure 2A). Similarly, we did not see any significant difference in the expression of *AXL* at the mRNA level (Figure 2B). While TYRO3 and MER proteins are not detectable by western blot in hCMEC/D3 cells, we found that the mRNA levels of *TYRO3* and *MER* were comparable in both media (Figure 2B).

We compared the transcriptome of hCMEC/D3 cells cultured in EBM-2 and in EndoGRO to identify cellular genes that could account for differences in susceptibility. Microarray analysis showed that the expression of most genes was left unchanged in the two conditions, at the exception of 101 genes, which were upregulated in cells cultured in EBM-2 as compared to cells cultured in EndoGRO, and of 38 genes that were upregulated in cells cultured in EndoGRO as compared to EBM-2 cultured cells (Figure 2C and Supplementary Tables 3 and 4).

Among the proteins expressed at higher level in EndoGRO medium than in EBM-2, we identified Claudin-7 (CLDN7). This gene caught our attention as claudins have been identified as modulators of cellular sensitivity to viruses from the *Flaviviridae* family (Colpitts and Baumert, 2017). The endogenous protein expression levels of CLDN7 could not be detected at the protein level in endothelial cells, as previously reported (Vu et al., 2009; Supplementary Figure 3). It was, however, detected in A549 and HEK 293T epithelial cells. Of note, CLDN7 could only be detected when analyzing whole cell lysates (i.e., non-centrifuged, comprising the insoluble fraction). RT-qPCR analysis confirmed that *CLDN7* mRNA abundance increased in cells grown in EndoGRO when compared to cells cultured in EBM-2 (Figure 2D), consistent with the microarray analysis. In contrast, the mRNA levels of *CLDN5* and *CLDN3* – the other predominant claudins expressed in hCMEC/D3 cells (Weksler et al., 2005; Vu et al., 2009; Schrade et al., 2012; Urich et al., 2012) – were unchanged. Of note, at the protein level, *CLDN5* expression was comparable in the different conditions (Figure 2A).

### Downregulation of *Claudin*-7 Reduces hCMEC/D3 Susceptibility to ZIKV Infection

We hypothesized that the ability of ZIKV to replicate more efficiently in cells grown in the EndoGRO medium, as compared to cells grown in EBM-2, could be (at least partially) due to differences in *CLDN7* expression levels.

To test the impact of *CLDN7* knockdown (KD) on hCMEC/D3 susceptibility to African ZIKV infection, hCMEC/D3 cells were transduced with lentiviral vectors expressing shRNA sequences targeting *CLDN7* or, as controls, *CLDN5* transcripts or non-targeting shRNAs (CTRL). Transduced cells were selected over 2weeks by puromycin treatment. The obtained cultures were a mix of populations of transduced cells.

As CLDN7 cannot be detected by western blot in hCMEC/D3 cells (Supplementary Figure 3), Knockdown was validated by RT-qPCR on cellular mRNA (Supplementary Figure 4). As half-life of claudins is short (6–12h; Van Itallie et al., 2019), one can assume that a significant reduction in claudin mRNA leads to reduced protein levels. Of note, *CLDN5* seems consistently overexpressed in the *CLDN7*-KD cells (Supplementary Figure 4).

**Figure 3:**
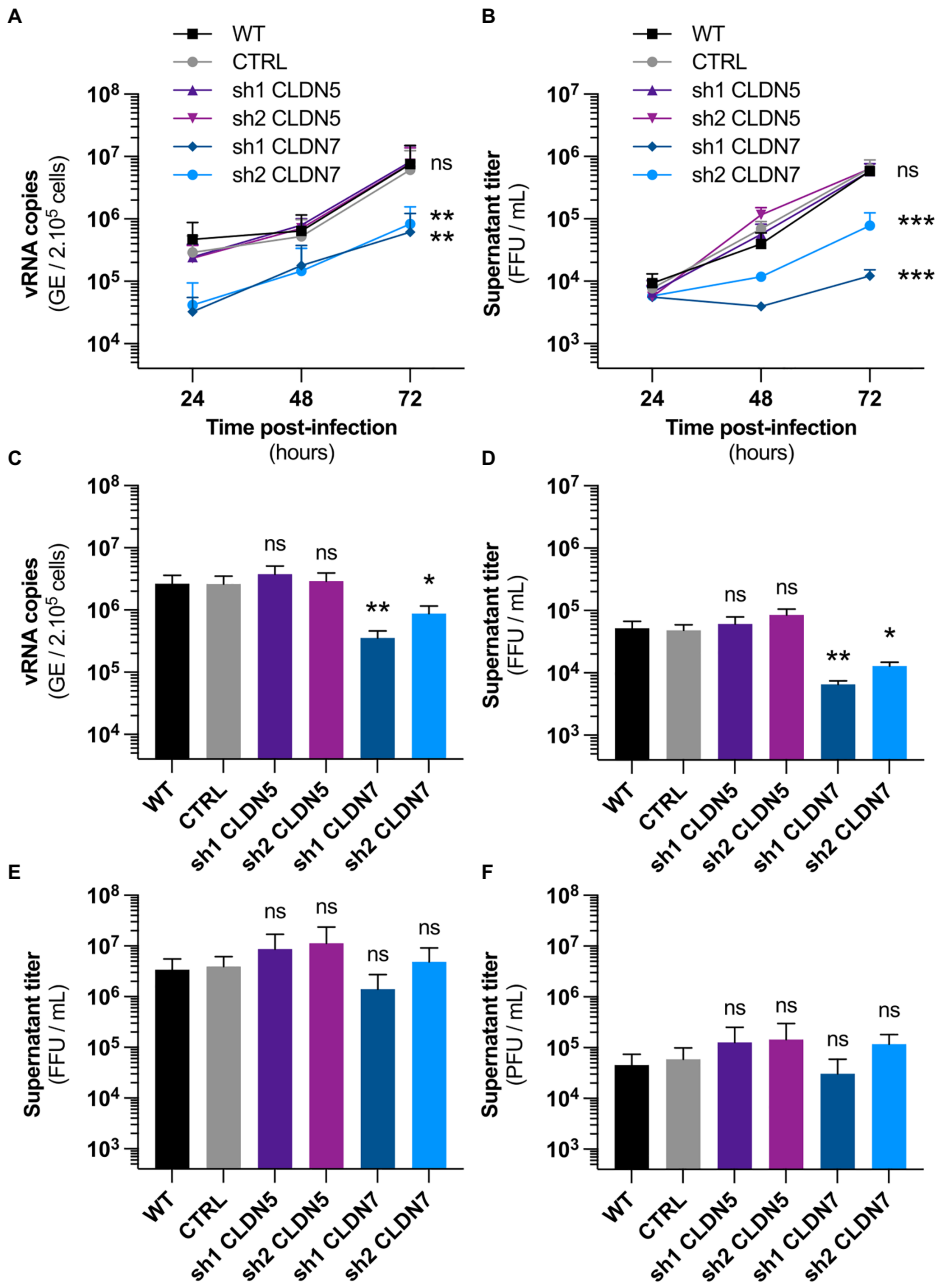
CLDN7-Knockdown alters hCMEC/D3 susceptibility to ZIKV infection. The hCMEC/D3 cells were transduced with lentivectors containing shRNAs targeting CLDN5 and CLDN7 transcripts or a non-targeting control shRNA (CTRL). **(A)** Time-course quantification of viral RNA through RT-qPCR in transduced hCMEC/D3 cells infected with HD78788 ZIKV at a MOI of 1 [*n*=3 independent experiments, *p*=0.03 (2-Way ANOVA, Sidak’s *post-hoc*)]. **(B)** Time-course quantification of viral production in the supernatant in transduced hCMEC/D3 cells infected with HD78788 ZIKV at a MOI of 1 [*n*=3 independent experiments, *p*=0.01 (2-Way ANOVA, Sidak’s *post-hoc*)]. **(C)** Quantification at 72h post-infection of viral RNA through RT-qPCR in transduced hCMEC/D3 cells infected with INMI1 ZIKV at a MOI of 1 [*n*=6 independent experiments, *p*<0.0001 (Friedman test, Dunn’s *post-hoc*)]. **(D)** Quantification at 72h post-infection of viral production in the supernatant in transduced hCMEC/D3 cells infected with INMI1 ZIKV at a MOI of 1 [*n*=6 independent experiments, *p*<0.0001 (Friedman test, Dunn’s *post-hoc*)]. **(E)** Quantification at 48h post-infection of viral production in the supernatant in transduced hCMEC/D3 cells infected with RP9 JEV at a MOI of 1 [*n*=3 independent experiments, *p*=0.07 (Friedman test, Dunn’s *post-hoc*)]. **(F)** Quantification at 48h post-infection of viral production in the supernatant in transduced hCMEC/D3 cells infected with 17D YFV at a MOI of 1 [*n*=3 independent experiments, *p*<0.001 (Friedman test, Dunn’s *post-hoc*)]. Each experiment was performed in triplicate. Data are presented as mean±SEM. ^***^p≤0.001; ^**^p≤0.01; ^*^p≤0.05; ns, *p*>0.05 (*post-hoc* corrected value of *p*). See also Supplementary Figure 4.

Transduction *per se* had no significant impact on hCMEC/D3 susceptibility (i.e., production of viral RNA and infectious particles) to ZIKV since hCMEC/D3 cells transduced with a lentiviral vector expressing a control shRNA (CTRL) were as susceptible as non-transduced (WT) hCMEC/D3 cells (Figures 3A,B). KD of *CLDN5* did not alter viral replication and production (Figures 3A,B). In contrast, viral replication and production were significantly reduced in *CLDN7*-KD cells, as compared to control cells (Figures 3A,B). *CLDN7* expression was also required for optimal replication of the Brazilian strain of ZIKV (Figures 3C,D), as shown by RT-qPCR analysis.

Together these data demonstrate that *CLDN7* expression favors ZIKV infection in hCMEC/D3 cells. Importantly, Japanese encephalitis and yellow fever viruses, both closely related to ZIKV, were replicating as efficiently in control hCMEC/D3 cells than in *CLDN7*-KD cells (Figures 3E,F). This demonstrates that *CLDN7* is specifically involved in hCMEC/D3 susceptibility to ZIKV infection.

### *Claudin-7* KD Reduced Susceptibility to ZIKV Infection of Endothelial Cells, but Not Epithelial Cells

We evidenced the contribution of *CLDN7* in ZIKV replication in hCMEC/D3 cells. We examined whether *CLDN7* was also favoring ZIKV infection in two other endothelial cell lines: bone marrow endothelial cells (TrHBMEC) and umbilical vein endothelial cells (HUVEC). We also tested the effect of silencing *CLDN7* expression in two epithelial cell lines: alveolar basal cells (A549) and embryonic kidney cells (HEK 293T).

Knockdown efficiency was validated by RT-qPCR in these four cell lines (Supplementary Figures 5A–D). We considered only cells with a −∆∆*C*_T_ (compared to WT cells) lower than −2; for HUVEC and HEK 293T, only one shRNA-derived cell line (out of five tested) was considered for further analysis (Supplementary Figures 5A–D). For epithelial cells, downregulation was validated at the protein level by western blot analysis (Supplementary Figures 5E,F).

**Figure 4:**
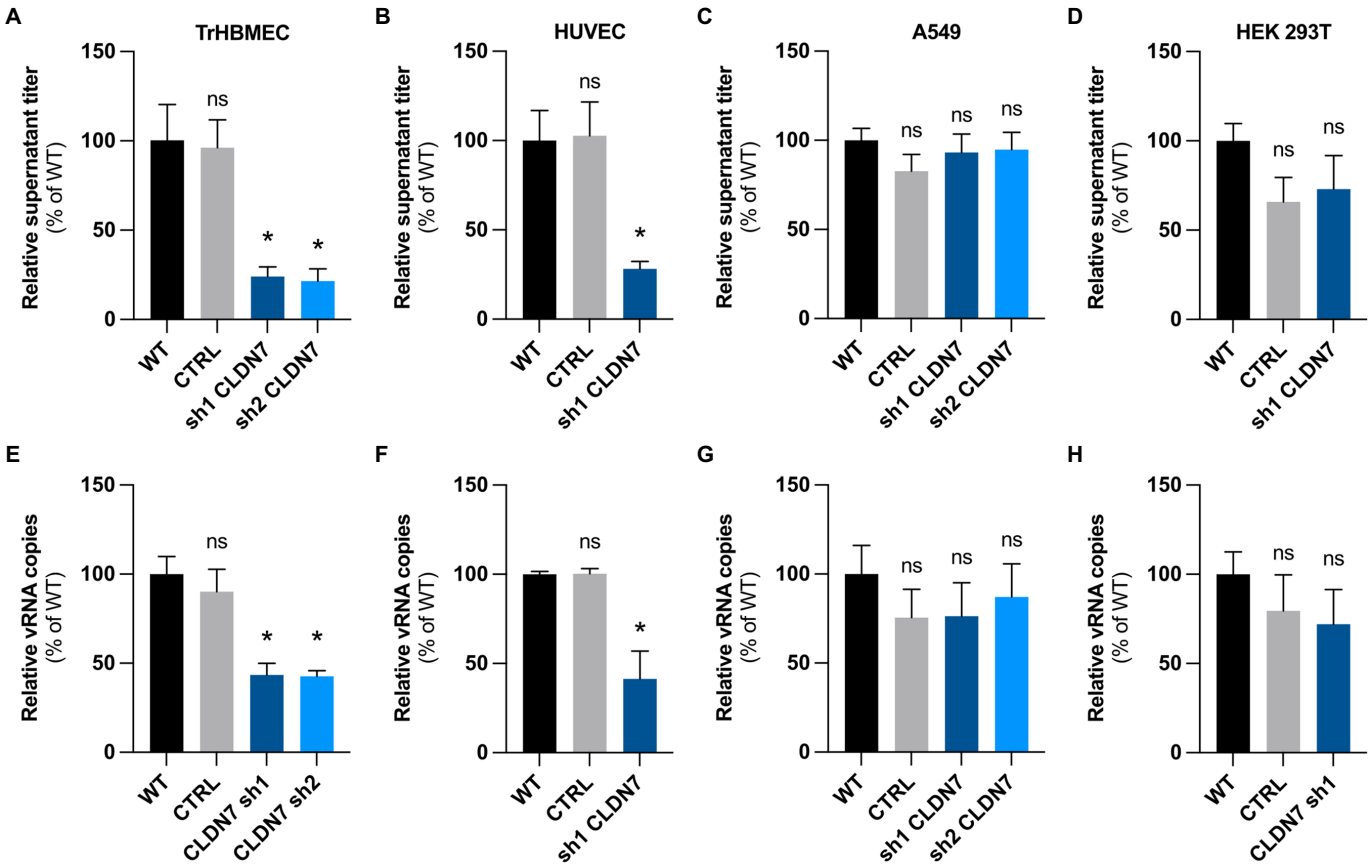
CLDN7-Knockdown alterations are limited to endothelial cells. TrHBMEC, HUVEC, A549, and HEK 293T cells were transduced with lentivectors containing shRNAs targeting CLDN7 transcripts or a non-targeting control shRNA (CTRL). **(A)** Relative quantification at 48h post-infection of viral production in the supernatant in transduced TrHBMEC cells infected with HD78788 ZIKV at a MOI of 1 [*n*=3 independent experiments, *p*=0.011 (Friedman test, Dunn’s *post-hoc*)]. **(B)** Relative quantification at 48h post-infection of viral production in the supernatant in transduced HUVEC cells infected with HD78788 ZIKV at a MOI of 1 [*n*=3 independent experiments, *p*=0.0087 (Friedman test, Dunn’s *post-hoc*)]. **(C)** Relative quantification at 48h post-infection of viral production in the supernatant in transduced A549 cells infected with HD78788 ZIKV at a MOI of 1 [*n*=3 independent experiments, *p*>0.05 (Friedman test, Dunn’s *post-hoc*)]. **(D)** Relative quantification at 48h post-infection of viral production in the supernatant in transduced HEK 293T cells infected with HD78788 ZIKV at a MOI of 1 [*n*=3 independent experiments, *p*>0.05 (Friedman test, Dunn’s *post-hoc*)]. **(E)** Relative quantification at 48h post-infection of viral replication in transduced TrHBMEC cells infected with HD78788 ZIKV at a MOI of 1 [*n*=3 independent experiments, *p*=0.005 (Friedman test, Dunn’s *post-hoc*)]. **(F)** Relative quantification at 48h post-infection of viral replication in transduced HUVEC cells infected with HD78788 ZIKV at a MOI of 1 [*n*=3 independent experiments, *p*=0.045 (Friedman test, Dunn’s *post-hoc*)]. **(G)** Relative quantification at 48h post-infection of viral replication in transduced A549 cells infected with HD78788 ZIKV at a MOI of 1 [*n*=3 independent experiments, *p*>0.05 (Friedman test, Dunn’s *post-hoc*)]. **(H)** Relative quantification at 48h post-infection of viral replication in transduced HEK 293T cells infected with HD78788 ZIKV at a MOI of 1 [*n*=3 independent experiments, *p*>0.05 (Friedman test, Dunn’s *post-hoc*)]. Each experiment was performed in triplicate. Data are presented as mean±SEM. ^*^p≤0.05; ns, *p*>0.05 (*post-hoc* corrected value of *p*). See also Supplementary Figure 4.

We found that downregulation of *CLDN7* significantly reduced infectious particle production in both endothelial cell types, TrHBMEC (Figure 4A) and HUVEC (Figure 4B). In contrast, no significant difference in viral yield was observed upon CLDN7-KD in A549 (Figure 4C) and HEK 293T epithelial cells (Figure 4D). Likewise, viral RNA production was significantly reduced in *CLDN7*-KD endothelial cells as compared to control cells (Figures 4E,F) but not in epithelial cells (Figures 4G,H).

Our data demonstrate that CLDN7 favors ZIKV replication in endothelial cells but not in epithelial cells.

### Claudin-7 Is Not Involved in the Early Steps of the Viral Cycle

**Figure 5:**
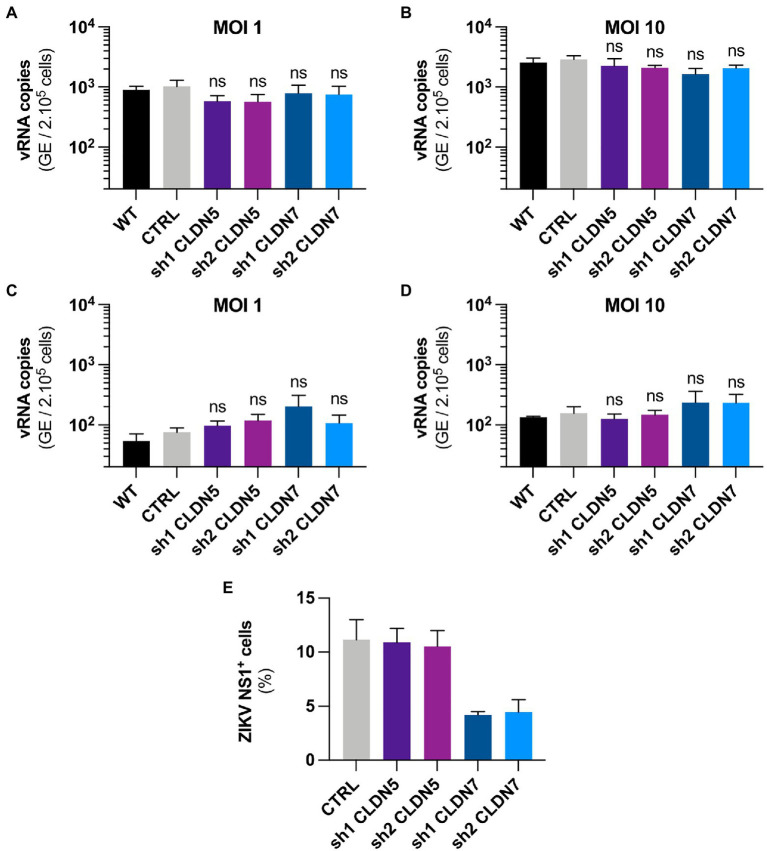
CLDN7-Knockdown does not alter the early steps of ZIKV cycle. The hCMEC/D3 cells were transduced with lentivectors containing shRNAs targeting CLDN5 and CLDN7 transcripts or a non-targeting control shRNA (CTRL). **(A,B)** Binding of HD78788 ZIKV to transduced hCMEC/D3 cells, quantification of viral RNA through RT-qPCR in cells culture with the virus for 1h at 4°C and extensively washed. **(A)** Cells were infected at a MOI of 1 [*n*=3 independent experiments, *p*>0.05 (Friedman test, Dunn’s *post-hoc*)]. **(B)** Cells were infected at a MOI of 10 [*n*=5 independent experiments, *p*>0.05 (Friedman test, Dunn’s *post-hoc*)]. **(C,D)** Internalization of HD78788 ZIKV in transduced hCMEC/D3 cells, quantification of viral RNA through RT-qPCR in cells from the binding assay furthermore kept for 2h at 37°C followed by removal of un-internalized virions. **(C)** Cells were infected at a MOI of 1 [*n*=3 independent experiments, *p*>0.05 (Friedman test, Dunn’s *post-hoc*)]. **(D)** Cells were infected at a MOI of 10 [*n*=3 independent experiments, *p*>0.05 (Friedman test, Dunn’s *post-hoc*)]. **(E)** Transduced hCMEC/D3 cells were infected with a MOI of 1 and kept for 16h to allow the virus to complete one replication cycle. Cells were stained with anti-NS1 antibodies and DAPI. ZIKV NS1-positive hCMEC/D3 cells were quantified (*n*=2 independent experiments). Each experiment was performed in triplicate. Data are presented as mean±SEM. ns, *p*>0.05 (*post-hoc* corrected value of *p*).

We next assessed which step of the viral cycle required the expression of CLDN7. To determine whether CLDN7 could be involved in viral adsorption to the cell membrane, hCMEC/D3 cells were infected with ZIKV for 1h at 4°C, which allows viral binding but prevents virus internalization. Unbound viruses were then removed through extensive washing. ZIKV RNA levels, corresponding to virions that remained attached to cell membranes, were quantified by RT-qPCR analysis. As expected, more viral particles were bound to the cells when incubated at a MOI of 10 than at a MOI of 1 (Figures 5A,B). However, no difference in viral RNA yield was detected between WT and *CLDN7*-KD cells, suggesting that CLDN7 is dispensable for ZIKV binding to cell membranes.

We next tested whether CLDN7 was involved in virus internalization. Following a 1h incubation at 4°C, cells were cultured at 37°C for 2h. Cells were treated with trypsin to remove non-internalized viral particles. Quantification of intracellular ZIKV RNA present in the cells, representing internalized particles, showed no significant difference between the control cells and *CLDN7*-KD hCMEC/D3 cells at MOI 1 and 10 (Figures 5C,D), suggesting that CLDN7 does not contribute to ZIKV internalization.

**Figure 6:**
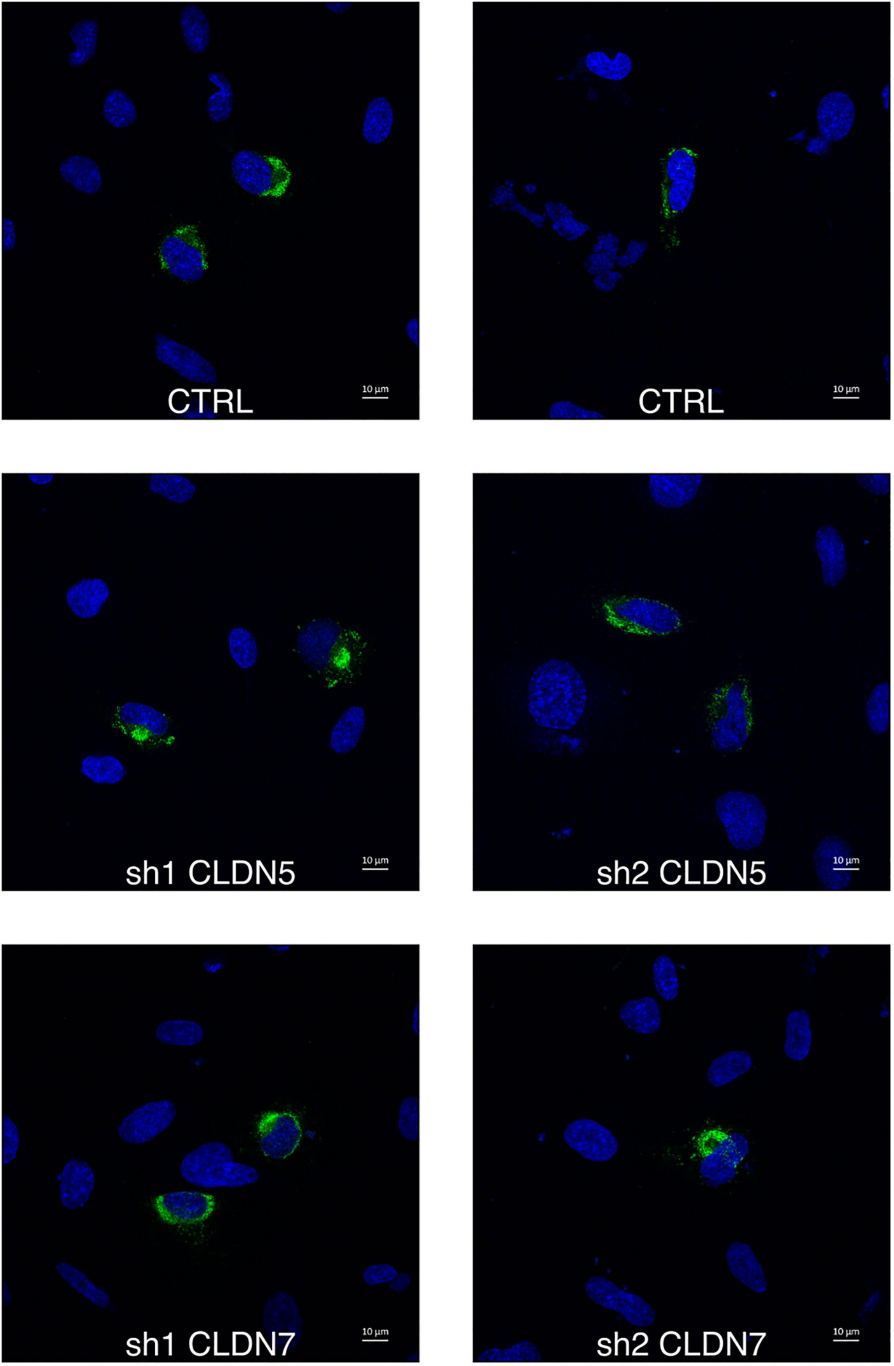
CLDN7-Knockdown does not alter ZIKV infection phenotype. The hCMEC/D3 cells were transduced with lentivectors containing shRNAs targeting CLDN5 and CLDN7 transcripts or a non-targeting control shRNA (CTRL). Transduced hCMEC/D3 cells were infected with ZIKV at a MOI of 1 and kept for 16h to allow the virus to complete one replication cycle. Confocal analysis of cells stained with anti-NS1 antibodies (green) and DAPI (blue). Representative images of ZIKV infected hCMEC/D3 cells.

The proportion of infected hCMEC/D3 cells was determined using staining against the viral non-structural protein NS1 at 16h post-infection, and a time that corresponds to a unique replication cycle (Figures 5E, 6). At a MOI of 1, around 11% hCMEC/D3 cells were expressing the NS1 protein. In contrast, in *CLDN7*-KD cells, only around 4% of cells were positive for NS1, independently of the shRNA that was used. These data confirm our previous RT-qPCR and titration analyses (Figure 3) and thus further validate the role of CLDN7 as a proviral factor in endothelial cells. Of note, the distribution of NS1 was comparable in infected WT and CLDN7-KD cells (Figure 6).

Together these results demonstrate that *CLDN7* downregulation does not affect viral attachment or internalization but reduces the number of cells expressing the viral protein NS1, suggesting that CLDN7 facilitates viral replication at a post-entry stage but prior to viral protein translation.

## Discussion

Since its emergence in the Pacific Islands and the Americas in 2015, ZIKV is classified as a neurotropic virus. Numerous reports have evidenced its neuroinvasion and neurovirulence abilities. The access of ZIKV to the CNS purportedly requires productive infection of BBB endothelial cells (Liu et al., 2010; Mladinich et al., 2017; Papa et al., 2017; Cle et al., 2020).

We confirmed that human BBB endothelial cells are susceptible to ZIKV infection (Liu et al., 2010; Mladinich et al., 2017; Papa et al., 2017; Cle et al., 2020). There are two commercially available media for the culture of cerebral endothelial cell. Intriguingly, we found that hCMEC/D3 cells were more susceptible to viral infection when cultured in EndoGRO than in EBM-2. We found that differences in susceptibility are likely due to medium-induced changes in the expression profile of hCMEC/D3. Microarray analysis revealed that cells cultured in EndoGRO expressed significantly more *CLDN7* than cells grown in EBM-2.

Although the complete composition of the media is not available, we know that EBM-2 is supplemented with bFGF while EndoGRO is supplemented with rhEGF. As previous papers have shown that epithelial cells overexpress CLDN7 upon EGF stimulation (Borok et al., 1996; Chen et al., 2005; Ferretti et al., 2017), we could hypothesize that CLDN7 overexpression may be, at least partially, due to the presence of rhEGF in EndoGRO. However, the transcriptomic differences (Supplementary Tables 3 and 4) cannot be fully attributed to the differences between EGF and FGF signaling: functional enrichment analysis using the software FunRich 3.1.3 did not evidenced a significant EGF-related signature. Other elements in the media likely participate in media-induced transcriptomic differences.

As reported in other studies, we found that CLDN7 protein expression was not detectable using western blot analysis and commercially available antibodies (Vu et al., 2009). Thus, we used CLDN7 mRNA abundance as a proxy for *CLDN7* expression. Stable shRNA-mediated CLDN7 downregulation impaired ZIKV replication in hCMEC/D3 cells and two other endothelial cell lines (i.e., HUVEC and HBMEC cells). However, the differential expression of *CLDN7* may not solely explain the differences in susceptibility observed between the cells grown in the two media. Indeed, the replication curves between CLDN7-KD cells and cells cultured in EBM-2 are not comparable at a MOI of 1 (Figure 1A vs. Figure 3A). Other cellular factors (as detected in the microarray) are thus likely to be at play and contribute to the differences in viral susceptibility.

The importance of claudins in the infection of other members of the *Flaviviridae* family has been previously reported (Colpitts and Baumert, 2017). In particular, CLDN1 is a major entry receptor for both HCV and dengue viruses in hepatocytes (Evans et al., 2007). Claudins form a large family of 24 members that are differentially expressed across tissues (Krause et al., 2008). There could be some redundancy between claudins as viruses might be able to interact with a variety of them. For instance, HCV can use CLDN6 and CLDN9 as alternative receptors to CLDN1 (Meertens et al., 2008). Such redundancy may explain why KD of CLDN7 in epithelial cells had no impact on viral replication. These cells express many claudins, which may substitute CLDN7. The nature of the claudins used by ZIKV may thus be cell-type or tissue specific.

Viral infection is carried out through several distinct steps. First, the virus is adsorbed at the cell surface, which is mediated by the interaction of the viral envelope with HSPG or C-type lectin receptors, such as DC-SIGN (Hamel et al., 2015; Kim et al., 2017). Different internalization paths have been proposed for ZIKV. For instance, the AXL-dependent internalization would mimic the internalization of apoptotic bodies (Meertens et al., 2017). Internalization could also occur upon usage of integrins (Wang et al., 2020), sialic-acids (Tan et al., 2019), or HSP70 (Pujhari et al., 2019). It is unclear whether viruses that exploit these different receptors end up in the same endosomal compartment or whether their fate depends on receptor usage, as suggested for related yellow fever viruses (Fernandez-Garcia et al., 2016). We show that CLDN7-KD does not affect ZIKV adsorption or internalization in endothelial cells.

By immunofluorescence analysis, we found that NS1 protein localization was comparable between CTRL and *CLDN7*-KD infected cells. This suggests that CLDN7 is not necessary for the establishment of effective replication sites in the cell. Indeed, if CLDN7 was part of the replication site, we would expect alterations in their perinuclear organization, as found for instance for Atlastin-3 (Monel et al., 2019).

Our data point to a role of CLDN7 anywhere between internalization and RNA production. It could for instance have a direct role in the fusion of the viral envelope with the endosomal membrane, as evidenced for occludin in the context of HCV infection (Benedicto et al., 2009). It could also have a more indirect function, such as regulating viral trafficking within endosomal compartments. Unfortunately, subcellular colocalization of CLDN7 and the virus could not be investigated in our system, as endogenous CLDN7 was not detectable by immunofluorescence.

In conclusion, we have found that CLDN7 is a susceptibility factor to ZIKV infection in endothelial cells. This finding provides functional insights into the mechanisms leading to ZIKV susceptibility in human endothelial cells *in vivo* and may be subsequently used as a novel target for antiviral drug development to prevent BBB disruption associated with ZIKV-related neurological disorders.

## Data Availability Statement

The datasets presented in this study can be found in online repositories. The names of the repository/repositories and accession number(s) can be found in the article/Supplementary Material.

## Author Contributions

JZ, VL, and PA conceived the study. P-EC, AG, and PA obtained funding. JZ, PJ, MC, and PA performed experiments. VL and NP provided viruses. JZ and PA wrote the manuscript. All authors contributed to the article and approved the submitted version.

## Funding

This work was supported by a Pasteur-Fiocruz grant and the “Investissement d’Avenir” as part of a “Laboratoire d’Excellence” (LabEx) French research program: Integrative Biology of Emerging Infectious Diseases (ANR10-LBX-62 IBEID). JZ was the recipient of a PhD fellowship from the ED562 – BioSPC and French ministry of education and research. VL was supported by a fellowship from the “Fondation pour la Recherche Médicale” (FRM).

## Conflict of Interest

The authors declare that the research was conducted in the absence of any commercial or financial relationships that could be construed as a potential conflict of interest.

## Publisher’s Note

All claims expressed in this article are solely those of the authors and do not necessarily represent those of their affiliated organizations, or those of the publisher, the editors and the reviewers. Any product that may be evaluated in this article, or claim that may be made by its manufacturer, is not guaranteed or endorsed by the publisher.
