# Supplementary material for: Zika Virus Requires the Expression of Claudin-7 for Optimal Replication in Human Endothelial Cells
Source: Front Microbiol. 2021 Sep 20;12:746589. doi: 10.3389/fmicb.2021.746589 (PMC8488266; doi:10.3389/fmicb.2021.746589)
Supplement: Supplementary file 1 [file Data_Sheet_1.PDF]

Supplementary Material

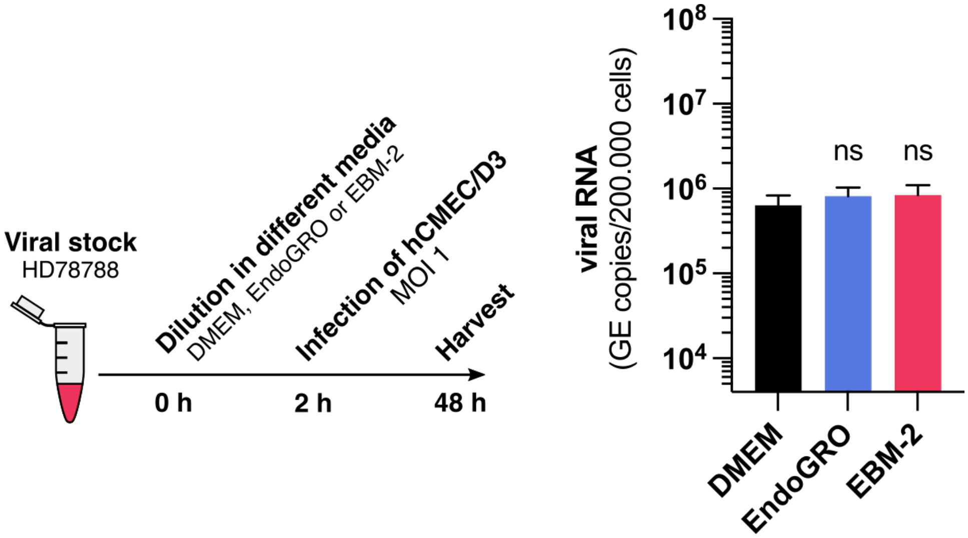

Supplementary Figure 1 – Medium does not affect intrinsic ZIKV infectiosity

A vial of the African HD78788 ZIKV was diluted in either DMEM, or EndoGRO, or EBM-2 for 2 h and was used to infect hCMEC/D3 cells. After viral adsorption (2h long), hCMEC/D3 cells were washed and cultured for 48 h. Viral infection and replication was then quantified RT-qPCR (n = 3 independent experiments, P > 0.05 [Kruskal-Wallis test, Dunn's *post-hoc*]).

Each experiment was performed in triplicate. Data are presented as mean ± SEM. ns, P > 0.05 (post-hoc corrected P-value).

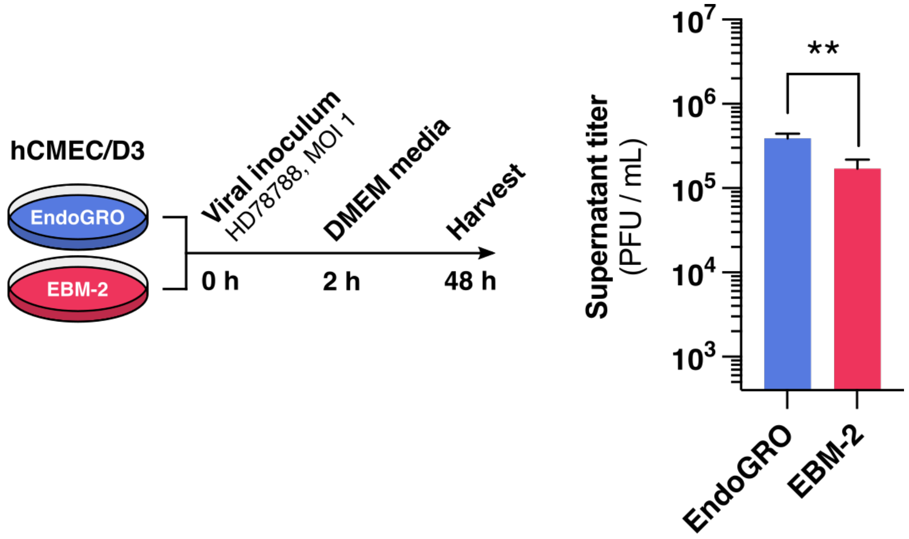

Supplementary Figure 2 – Culture medium prior to infection affects ZIKV susceptibility of hCMEC/D3 cells

hCMEC/D3 cells were cultured in EndoGRO or EBM-2 and infected with ZIKV (African HD78788 strain) resuspended in DMEM. After adsorption, the cells were kept in DMEM for 48 h. The supernatant was collected and the viral titer was quantified by plaque assay (n = 3 independent experiments, [Mann-Whitney t-test]).

Each experiment was performed in triplicate. Data are presented as mean ± SEM. **$P \leq 0.01$.

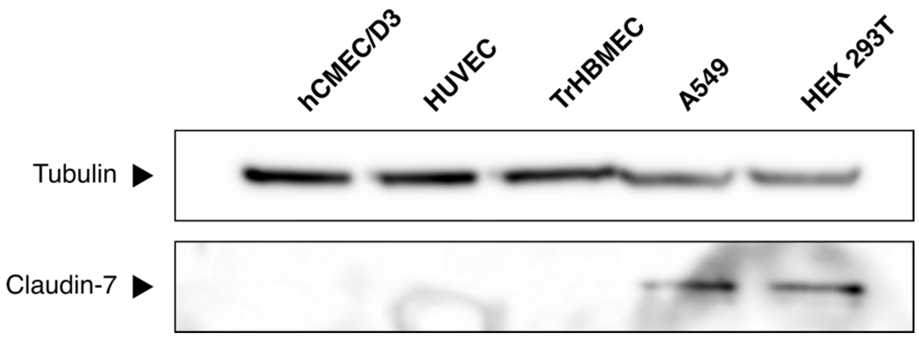

Supplementary Figure 3 – Claudin-7 expression in different cell lines

Western blot analysis of claudin-7 expression in several endothelial (hCMEC/D3, HUVEC and TrHBMEC, all cultivated in EndoGRO) and epithelial (A549, HEK 293T) cells. α-Tubulin serves as loading control.

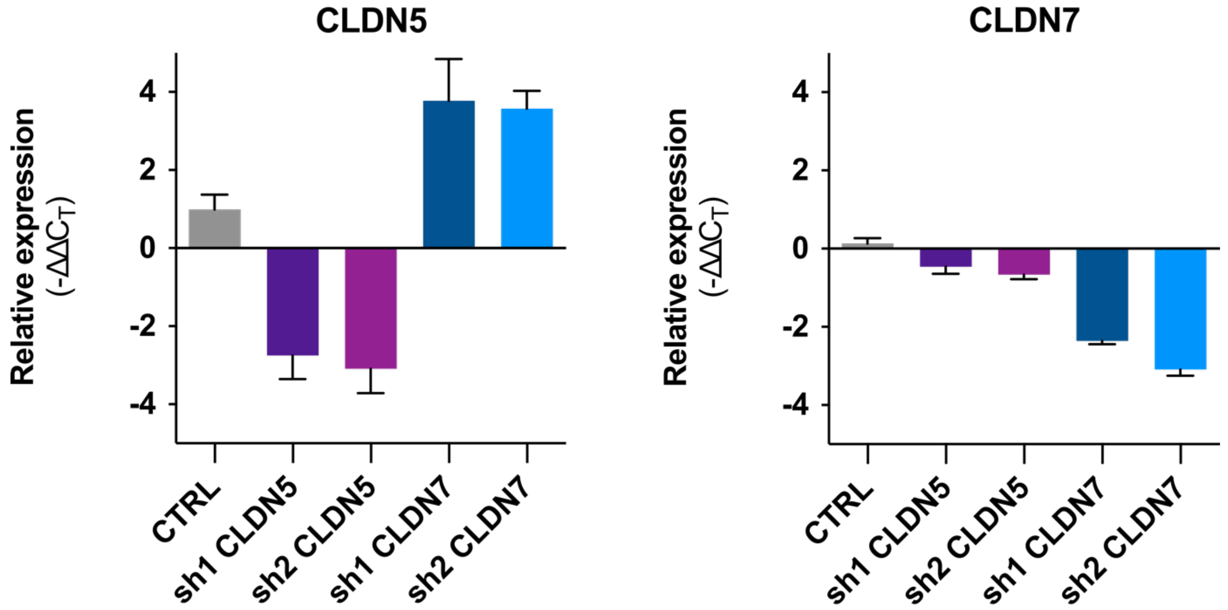

Supplementary Figure 4 – Knockdown efficiency in hCMEC/D3 cells

Differential mRNA levels for CLDN5 (left) and CLDN7 (right) through RT-qPCR in hCMEC/D3 cells transduced with shRNA targeting CLDN5 and CLDN7 transcripts or non-targeting (CTRL) relative to non-transduced cells (n = 3 independent experiments).

Each experiment was performed in triplicate. Data are presented as mean ± SEM.

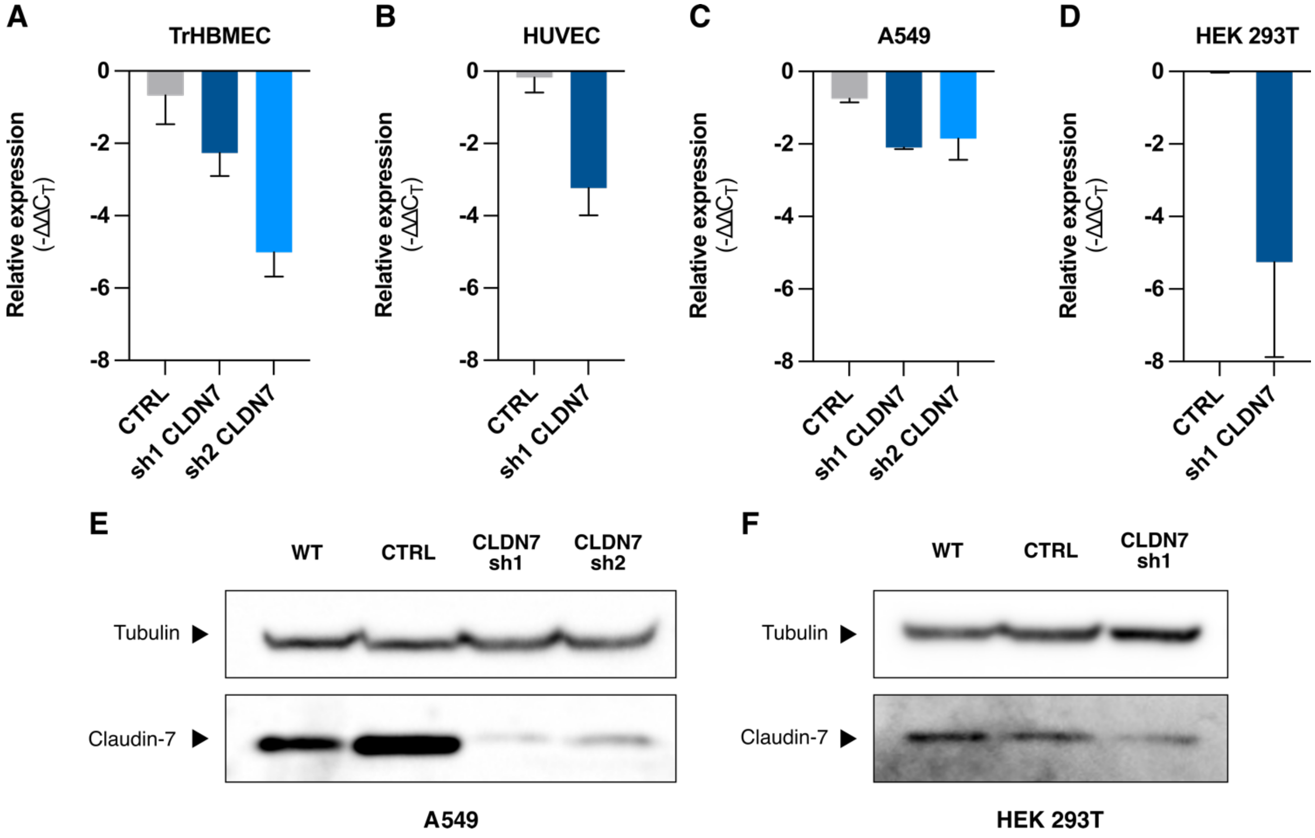

Supplementary Figure 5 – Knockdown efficiency in TrHBMEC, HUVEC, A549 and HEK 293T cells

(A) Differential mRNA levels for CLDN7 through RT-qPCR in TrHBMEC cells transduced with shRNA relative to non-transduced cells (n = 3 independent experiments).

(B) Differential mRNA levels for CLDN7 through RT-qPCR in HUVEC cells transduced with shRNA relative to non-transduced cells (n = 3 independent experiments).

(C) Differential mRNA levels for CLDN7 through RT-qPCR in A549 cells transduced with shRNA relative to non-transduced cells (n = 3 independent experiments).

(D) Differential mRNA levels for CLDN7 through RT-qPCR in HEK 293T cells transduced with shRNA relative to non-transduced cells (n = 3 independent experiments).

(E) Western blot analysis of claudin-7 expression in A549 cells transduced with shRNA. α-Tubulin serves as loading control.

(F) Western blot analysis of claudin-7 expression in HEK 293T cells transduced with shRNA. α-Tubulin serves as loading control.

Each experiment was performed in duplicate. Data are presented as mean ± SEM.

Supplementary Table 1 – List of RT-qPCR primers

**Target gene**	**Sequence** (5' – 3')	**Amplicon size**
AXL	F: GTGGGCAACCCAGGGAATATC<br>R: GTACTGTCCCGTGTCGGAAAG	234 bp
CLDN3	F: CTGCTCTGCTGCTCGTGTCC<br>R: TTAGACGTAGTCCTTGCGGTCG	129 bp
CLDN5	F: GACTCGGTGCTGGCTCTGAG<br>R: CGTAGTTCTTCTTGTCGTAG	451 bp
CLDN7	F: AGGCATAATTTTCATCGTGG<br>R: GAGTTGGACTTAGGGTAAGAGCG	252 bp
GAPDH	F: GGAGCGAGATCCCTCCAAAAT<br>R: GGCTGTTGTCATACTTCTCATGG	197 bp
MER	F: CAGGAAGATGGGACCTCTCTGA<br>R: GGCTGAAGTCTTTCATGCACGC	115 bp
TYRO3	F: GTGTGTGGCTGACTTCGGAC<br>R: CACGTCCTCCATACACTCCG	277 bp
ZIKV *NS5*	F: AAGTACACATACCAAAACAAAGTG<br>R: TCCGCTCCCCCTTTGGTCTTG	101 bp

Supplementary Table 2 – List of antibodies used in western blots

Target	Type of antibody	Manufacturer	Reference	Dilution
AXL	Rabbit Primary	Cell Signal	C89E7	1:1000
Claudin-5	Rabbit Primary	Abcam	AB15106	1:1000
Claudin-7	Rabbit Primary	Merck	SAB4500437	1:500
Rabbit IgG	HRP-conjugated Secondary	Merck	NA9340	1:5000
α-Tubulin	Mouse Primary	Merck	T5168	1:4000
Mouse IgG	HRP-conjugated Secondary	Thermofisher	31430	1:5000

Supplementary Table 3 – List of upregulated genes in hCMEC/D3 cells cultivated in EndoGRO

Gene	Fold-change	P-value
PHLDA2	2,00047521	3,344E-07
AGMAT	2,02427494	2,345E-05
SSBP4	2,02433878	2,978E-05
PCMTD2	2,03883779	6,103E-05
MIOS	2,04929723	1,090E-06
C2orf81	2,06305173	1,169E-06
LOXL1	2,14953299	5,385E-05
CDK6	2,15942132	2,196E-05
CCDC71L	2,27734087	3,570E-05
MYO1B	2,34501069	2,263E-05
ADAM19	2,36186417	6,398E-06
NES	2,36205491	3,978E-07
CLDN7	2,36512224	7,688E-05
SULT1E1	2,39945624	5,640E-05
COL6A3	2,43517086	3,467E-06
DOCK10	2,44539454	2,817E-06
HIST1H2AK	2,49261316	9,600E-06
PSAT1	2,5226464	1,656E-06
SNCA	2,80139758	7,373E-05
SLC9A7	2,80370974	6,410E-05
ADAMTSL1	2,80376999	3,344E-05
SRPX	2,89524814	1,020E-06
LOC101927746	2,9097395	9,856E-05
NR2F1	2,99543858	6,041E-06
CLU	3,0482305	3,675E-05
LAMB3	3,16332711	3,597E-06
FAM49A	3,16585022	3,161E-05
MATN3	3,1948169	4,659E-05
POLR3G	3,66355303	1,575E-05
MMRN1	4,03713547	9,336E-06

CD200	4,20415249	2,571E-07
SCG2	4,36299554	9,238E-06
ANGPT2	5,09426144	1,379E-05
ETV4	5,12833498	6,427E-07
SCARA3	6,31706536	9,401E-08
CCL2	7,10237236	5,197E-05
PTGS1	7,58856317	5,796E-05
TRBV3-1	13,9133941	6,333E-05

Supplementary Table 4 – List of downregulated genes in hCMEC/D3 cells cultivated in EndoGRO

Gene	Fold-change	P-value
PLPP3	0,49676029	9,163E-05
STARD8	0,49599354	7,790E-06
NT5E	0,49232469	4,716E-06
FRMD3	0,48586747	4,461E-06
ST6GALNAC3	0,48452995	2,107E-05
TMEM38A	0,48066145	1,885E-05
HSD17B14	0,47905435	4,057E-06
BACE2	0,47697498	5,695E-05
BMPR2	0,47348972	9,440E-07
ABLIM1	0,47250132	7,314E-05
GCH1	0,46872812	2,392E-05
ABCC3	0,46485349	4,713E-08
MRC2	0,4560343	5,625E-05
DDX60L	0,45565088	6,006E-05
SNAPC1	0,45421224	3,573E-07
ARFGEF3	0,45352037	3,243E-06
SYNE2	0,45295205	3,701E-06
PON2	0,45229549	9,948E-09
MYO1D	0,45156779	5,326E-06
MIR3671	0,44943784	8,868E-05
NEGR1	0,44791038	6,032E-05
CYP27A1	0,4478864	9,773E-06
STOX1	0,4477554	2,857E-06
LYN	0,44747423	9,755E-06
TLR4	0,44695895	1,023E-06
LOC105376446	0,44683505	7,173E-05
MTMR10	0,44599734	4,100E-06
HEG1	0,44078888	8,596E-08
DNER	0,43691786	5,896E-07
AVPI1	0,43454583	1,390E-06

GSN	0,43451842	2,641E-06
GAS6	0,43373379	6,745E-06
C1orf198	0,43098035	5,419E-05
RALGAPA2	0,42165238	1,755E-06
SCNN1B	0,42105212	9,895E-05
CMAHP	0,42043808	6,257E-06
KLF11	0,4203841	4,081E-06
ARHGEF10	0,41866523	3,409E-06
APOA1-AS	0,41774863	2,283E-05
TMEM204	0,41449756	3,818E-05
GALNT15	0,40769644	4,575E-07
ANKH	0,40517245	6,496E-05
VLDLR	0,40478977	1,526E-05
GRAMD1B	0,40453481	3,117E-05
IGFBP3	0,40438251	3,400E-05
ASAH1	0,4030938	3,809E-06
HIF1A-AS2	0,38842642	6,692E-05
PLLP	0,38680444	6,427E-07
HAND1	0,38107536	3,155E-05
RPL23AP32	0,37609323	9,579E-05
SEL1L3	0,37473477	2,648E-07
WFDC1	0,37396464	2,999E-05
CEMIP	0,37132735	5,830E-05
RGS7	0,36522052	4,679E-05
NDRG4	0,36302964	1,009E-05
AIF1L	0,36194427	1,989E-07
ADGRF5	0,3606681	2,207E-06
HDAC5	0,35725287	1,749E-06
IL3RA	0,35586643	1,425E-07
BVES	0,35398607	9,231E-05
RAPGEF4	0,35391603	3,642E-06
ST8SIA6	0,35374999	5,543E-05
BMP6	0,35343217	1,493E-07

GAS6-AS2	0,34773628	7,419E-05
RTKN2	0,34039583	2,151E-05
CD34	0,34006678	1,310E-06
KLF4	0,33357123	1,875E-05
IL3RA	0,33206798	9,912E-08
HERC5	0,33142883	2,778E-05
TFRC	0,32429929	4,546E-06
LRRC16A	0,31794337	3,151E-05
PTPRG	0,31505132	7,878E-06
ASS1	0,31477426	1,516E-05
FOXC1	0,31187191	1,134E-05
ADRB2	0,3095872	1,601E-06
ZNF366	0,30534021	7,254E-07
PTPRB	0,29885534	7,657E-06
SLCO2A1	0,29381903	4,019E-05
LYPD1	0,28376223	2,132E-05
ST6GALNAC2	0,28359328	1,891E-05
PPP1R14A	0,28252477	1,338E-05
IFI44L	0,2738229	2,186E-05
TMEM45A	0,27291829	1,642E-07
HECW2	0,24651571	2,078E-05
HCN2	0,24504923	2,640E-06
LINC00520	0,23697151	2,137E-05
IRF6	0,23672139	1,681E-06
KCTD8	0,22474217	8,570E-06
CRLF1	0,22374235	5,841E-06
SMAD6	0,22262122	4,688E-06
CRYAB	0,21054886	2,522E-07
MYL2	0,21011233	2,555E-05
SORBS1	0,20120063	7,905E-08
INHBA	0,20105112	9,996E-07
CMPK2	0,19445106	7,866E-05
RSAD2	0,18875742	9,385E-05

NPR3	0,1722484	1,084E-05
SERPINE2	0,15364562	1,124E-05
SLPI	0,0789098	3,067E-06
RBP7	0,06052236	2,578E-07
ALPL	0,01723549	1,761E-07
